# Journey of vulnerability: a mixed-methods study to understand intrapartum transfers in Tanzania and Zambia

**DOI:** 10.1186/s12884-020-02996-8

**Published:** 2020-05-14

**Authors:** Tina Lavender, Carol Bedwell, Kieran Blaikie, Valentina Actis Danna, Chris Sutton, Chowa Tembo Kasengele, Sabina Wakasiaka, Bellington Vwalika, Rose Laisser

**Affiliations:** 1 Jean McFarlane Building, University of Manchester, Manchester, M139PL UK; 2 Ministry of Health Headquarters, Department of Public Health and Research, Lusaka, Zambia; 3 University of Nairobi, Nairobi, Kenya; 4 University of Zambia ,School of Medicine, Lusaka, Zambia; 5 Archbishop Antony Mayala School of Nursing, Catholic University of Health and Allied Health Sciences, Mwanza, Tanzania

**Keywords:** Transfers, Intrapartum, Mixed-methods, Qualitative, Grounded theory, Observation, Case note review, Tanzania, Zambia

## Abstract

**Background:**

Timely intrapartum referral between facilities is pivotal in reducing maternal/neonatal mortality and morbidity but is distressing to women, resource-intensive and likely to cause delays in care provision. We explored the complexities around referrals to gain understanding of the characteristics, experiences and outcomes of those being transferred.

**Methods:**

We used a mixed-method parallel convergent design, in Tanzania and Zambia. Quantitative data were collected from a consecutive, retrospective case-note review (target, *n* = 2000); intrapartum transfers and stillbirths were the outcomes of interest. A grounded theory approach was adopted for the qualitative element; data were collected from semi-structured interviews (*n* = 85) with women, partners and health providers. Observations (*n* = 33) of transfer were also conducted. Quantitative data were analysed descriptively, followed by binary logistic regression models, with multiple imputation for missing data. Qualitative data were analysed using Strauss’s constant comparative approach.

**Results:**

Intrapartum transfer rates were 11% (111/998; 2 unknown) in Tanzania and 37% (373/996; 1 unknown) in Zambia. Main reasons for transfer were prolonged/obstructed labour and pre-eclampsia/eclampsia. Women most likely to be transferred were from Zambia (as opposed to Tanzania), HIV positive, attended antenatal clinic < 4 times and living > 30 min away from the referral hospital. Differences were observed between countries. Of those transferred, delays in care were common and an increase in poor outcomes was observed. Qualitative findings identified three categories: *social threats to successful transfer*, *barriers to timely intrapartum care* and *reparative interventions* which were linked to a core category: *journey of vulnerability.*

**Conclusion:**

Although intrapartum transfers are inevitable, modifiable factors exist with the potential to improve the experience and outcomes for women. Effective transfers rely on adequate resources, effective transport infrastructures, social support and appropriate decision-making. However, women’s (and families) vulnerability can be reduced by empathic communication, timely assessment and a positive birth outcome; this can improve women’s resilience and influence positive decision-making, for the index and future pregnancy.

## Background

Intrapartum referrals between facilities are an essential part of obstetric management in low-income settings, aimed at ensuring that women get the appropriate emergency care in hospitals that have skilled birth attendants and adequate resources. Multiple reasons for transfer in labour exist; reflecting some of the major causes of maternal/neonatal mortality and morbidity and include obstructed labour, pre-eclampsia and haemorrhage [1].

Functional referral systems are an essential component of health systems [2] and should include timely assessments and decision-making, a pre-established referral plan, appropriate information exchange and feedback to relevant health care staff [3]. However, in low income regions, including sub-Saharan Africa, these sub-components are not always included. Researchers [4, 5] have demonstrated the impact of poor referral systems on high perinatal mortality rates, which undoubtedly contribute to the 4.8 million perinatal deaths annually; 98% of which occur in low and middle income settings [6]. Reasons proposed for poor referral systems include staff shortages, inadequate staff training, and lack of transport [7]; additionally, women themselves are sometimes reluctant to be referred [7].

The 3-delays model [8], historically used when researching maternal mortality [8], has been proposed as a framework for understanding the complexities related to intrapartum referral [4, 5, 9] and supporting insight into perinatal deaths. This model [8] enables exploration of delays in decision-making (delay 1), delays in a woman accessing care (delay 2) and delays in receiving timely, appropriate care/treatment (delay 3). Importantly, studies focussing on the 3-delays model have identified the importance of ‘supply’ and ‘demand’ interventions [10] to improve outcomes. This emphasises the need to explore referrals from multiple perspectives.

A major gap in the literature is the experiences of those involved in referrals in low-income settings. Drawing from high-income settings, particularly Australia, it is evident that women who are transferred to a referral facility suffer emotionally and often have to adapt to an environment in which they are unfamiliar [11]. Partners also find the process of transfer difficult; particularly having to observe the woman’s challenging journey [11]. Giving birth in a place that was unintended has, in itself, been reported as having an impact on a woman’s experience [12]. Although women acknowledge the expertise available at the referral facility [13], they can still remain disappointed at not birthing in their place of choice [12, 14]. Furthermore, some partners and health providers (from the referring facility) have stated that they believe that their views are not always valued [14].

Although one can gain valuable insight from high-income settings on experiences of intrapartum transfers, the resource-rich study context and the higher socio-economic status of participants makes it inappropriate to transfer the findings to countries in sub-Saharan Africa. Countries, such as Tanzania and Zambia, have complex challenges such as poor geographic access to emergency obstetric and neonatal care (EmONC) services, [15, 16] limited emergency transportation and insufficient skilled birth attendants [17, 18]. Thus access to quality evidence-based care remains problematic. This is compounded by the cultural influences on childbirth decision-making which have the potential to hinder referral processes [19].

Uniquely, we adopted a holistic approach to gain a comprehensive understanding of the complexities surrounding intrapartum referrals in Tanzania and Zambia. We examined the prevalence and reasons for transfer, the experiences of those involved and the outcomes of those transferred (and those not transferred, for comparison). The latter was important to confirm the extent to which these groups differed, gain understanding of how they differed and provide robust contextual information for the study settings.

## Methods

### Study setting and design

We conducted a mixed-method parallel convergent design, involving a retrospective case-note review, interviews and observations in rural and urban settings in Tanzania and Zambia. The case-note review took place in two tertiary referral hospitals; the Tanzania setting has 5662 births and Zambia 3200 births annually. The national stillbirth rates for Tanzania and Zambia are 21/1000 and 22/1000 live births respectively [20]. The interviews and observations took place in the referral, secondary and primary facilities to enable capture of views and experiences from different sites.

### Quantitative methods

#### Data collection

We conducted a consecutive, retrospective case-note review capturing births from July to September 2018. The duration of data collection was originally determined by the need to capture sufficient poor birth outcomes. The sample size calculation was based on the use of binary logistic regression to determine variables associated with a variety of key outcomes for the overall study, including intrapartum stillbirth or ‘near-miss’ [21] mortality, and intrapartum transfer. A sample size of 1700 with key data was chosen as this was considered sufficient to estimate a moderate number of parameters for multivariable binary logistic regression models to predict these outcomes: for intrapartum stillbirth or ‘near-miss’ mortality, with an estimated prevalence of 10%, this would enable estimation of 17 parameters (e.g. binary or continuous covariates), and for intrapartum transfer, with a transfer rate of 30%, this would enable estimation of 51 parameters, both based on the 10 ‘events per parameter’ guidance [22]. Allowing for missing data, we aimed to collect data from 1000 case notes from each country.

Women’s data were included if they had been admitted to the participating hospitals in the intrapartum period with a pregnancy of > 28 weeks gestation, during the study period, regardless of district of origin.

Case report forms (CRFs) were developed from WHO’s ICD-PM audit form [23] and adapted following input from the research team, stakeholders and community engagement and Involvement group (CEI). Forms were piloted on a sample of case records (*n* = 9 Tanzania; 6 Zambia) outside of the inclusion time frame.

Data were collected directly from the case records and birth register, by two trained research assistants in each country. Study data were collected and managed using Research Electronic Data capture tools (REDcap) [24] hosted at the institution of the lead author. In Tanzania, data were entered onto paper CRFs then transferred to REDCap. In Zambia, data were entered directly onto REDCap via mobile phones. To maximise accuracy and to minimise missing data error a random sample of records (10%) were double entered and discrepancies reviewed. The discrepancy rate was 0.8% Zambia and 1.0% Tanzania; these were corrected and no further action was required.

#### Analysis

Data were anonymised, with personal identifiers removed before analysis, then transferred into R Version 3.5.1 [25] for analysis. Descriptive statistics were produced outlining how population characteristics differed by country. Multivariable binary logistic regression models were produced to express the relationships between population characteristics and intrapartum transfer status. Characteristics to be included in the logistic regression models were chosen based on prior subject-matter expertise, with necessary inclusion of common confounders. Multiple imputation by chained equations (MICE) methods [26] were used to account for missing data, replacing missing observations with values we would expect, given the remaining information we know about each individual (i.e. under a ‘Missing At Random’ mechanism). This approach allowed us to use all the available information in our model for intrapartum transfer, thus retaining statistical power. We also produced separate models by country, based of multiply imputed data.

### Qualitative methods

#### Data collection

A grounded-theory methodology was adopted, informed by Symbolic Interactionism [27], to gain understanding of the impact of social interactions on participants’ views and experiences. Grounded theory enables one to move beyond descriptive accounts, enabling theory to emerge directly from the data [28]. The Straussian approach was used [29], chosen primarily because it adopts an iterative and inductive process.

Data were collected using interviews and observations. The inclusion of field notes, memos and multiple sources of data enabled understandings to be contextually grounded, through constant comparison. To ensure theoretical sensitivity [30], an open stance was aimed for and reflexivity maintained throughout.

In keeping with grounded theory, an initial purposive sample of 3 participants, in each country, of the following groups were recruited: postnatal women with a live birth, postnatal women with a stillbirth, postnatal women with a near miss mortality [21], partners of postnatal women, health professionals, and traditional birth attendants. All participants were at least 18 years old and were willing and able to consent.

#### Recruitment

The recruitment strategy was informed by the CEI group. Women were recruited by trained research assistants (midwives) in the hospital postnatal ward or follow-up clinic. Male partners were approached in the community, clinics or postnatal ward. Initial contact was made by a clinician, who notified the researcher only if permission to consider the study had been granted. Written and verbal information was supplied and potential participants given up to 4 weeks to consider participation. For those who showed an interest in participation, a ‘consent to contact form’ was completed so that the research assistant could contact them directly at a later date, using their preferred means, without the need for further interaction with the clinical care team. Written consent was obtained for those who agreed to participate. Traditional birth attendants (TBA) and Safe Motherhood Action Group Members (SMAG) were recruited through snowball sampling. Health professionals (nurse/midwives, ambulance drivers) were recruited from the local health facilities via posters, with contact details provided to enable opt-in to the study. After the initial purposive sample, data retrieval was directed by theoretical sampling, meaning that recruitment of additional participants was required for some groups and further groups needed targeting. The sample size was determined by data saturation.

#### Interviews

Participants were interviewed, by trained research assistants, in local language or English, depending on preference. Demographic details were collected at the beginning of the interview, to enable contextualisation of findings. These questions were interviewer-administered and incorporated socio-demographic, clinical and birth preparedness questions [31].

In-depth interviews were used to increase the authenticity of the data [32], as participants were encouraged to provide their own narratives, without the influence of others. In keeping with grounded theory [29], the topic guide, which was specifically designed for this study (Additional file 1) contained minimal questions to ensure that the interview was respondent-led. Each interview commenced with an opening question: *‘What are your thoughts about your pregnancy and birth?’* before conducting individualised deeper explorations. New insights were followed up in subsequent interviews for confirmability. Interview location was chosen by the participant and included home, community venue, hospital or clinic. Field notes captured any interview process nuances and incorporated non-verbal communications.

#### Observations

Non-participant observations were used to explore context, processes, behaviour and communications at the point of transfer. Observations enabled a “nuanced and dynamic” appreciation of transfers that could not be captured through other approaches [33]. Observations at each health facility level captured transfers to and from facilities. To avoid selectivity, researchers observed different circumstances, related to transfer, i.e. different times of the day, evening, during the week and weekend. It was not possible to obtain consent in advance of observations, as transfers were carried out in unanticipated emergencies. Prospective consent was also inappropriate due to women’s vulnerability and the potential to alter the dynamics of the observation. Thus, retrospective consent was obtained from those who were observed (woman, health professional, relative) soon after the transfer had taken place. The observation took a three-stage ‘funnel’ approach [34], beginning with descriptive observation to get an overview of the setting, moving to focused observation, to pay attention to a narrower portion of the transfer, and then selected observation, in which relationships were investigated. An observation grid, which was piloted and adapted prior to use, assisted with this process. Observations ended when theoretical saturation was reached, i.e. when no new understandings were emerging.

#### Analysis

The Grounded Theory approach, described by Strauss and Corbin [29], using three stages of coding was used: open, axial and selective. Five authors (TL, CB, RL, SW, CK) conducted the analysis, before seeking confirmation from the remaining authors. Following verbatim transcription, translation and back translation for confirmability, transcripts were read in their entirety for familiarisation and understanding. Line-by-line coding was carried out manually, whereby properties and dimensions were explored by systematically examining the whole document and its parts.

The axial coding involved the constant comparative technique, whereby comparisons were made between different transcripts and relationships identified. Codes were clustered into initial sub-categories, according to their commonalities, and were grouped. There were several iterations of categories, which were constantly reorganised to gain understanding of their meaning and their relationship to each other. The researchers moved between observations and interview data during the analysis. This process resulted in theoretical categories which in turn led to the core category (central concept).

## Results

### Quantitative

Characteristics differed by country, as well as the number of observations missing per variable (Table 1). The stillbirth rates were high; 16% in Tanzania and 10% in Zambia. Intrapartum transfers were less common in Tanzania (11%) compared to in Zambia (37%). The percentage of women who experienced at least one delay was greater in Tanzania than Zambia (66% vs 50%), although there was also a larger number (*n* = 348 vs. *n* = 27) from Zambia with missing delay data.

**Table 1:** Demographic details

Pregnancy Outcome Indicators	Both Countries	Tanzania	Zambia
Cases collected	1997	1000	997
- Maternal death (%)	35 (2%)	18 (2%)	17 (2%)
- Near-misses (%)	205 (17%) (812 NA)	70 (7%) (11 NA)	135 (69%) (801 NA)
- Prolonged Labour (%)	250 (13%) (8 NA)	48 (5%) (4 NA)	202 (20%) (4 NA)
- Reduced Foetal Movement (%)	195 (11%) (271 NA)	132 (13%) (6 NA)	63 (9%) (265 NA)
- Intrapartum Transfer (%)	484 (24%) (3 NA)	111 (11%) (2 NA)	373 (37%) (1 NA)
- Any Delay to Care (%)	963 (59%) (375 NA)	639 (66%) (27 NA)	324 (50%) (348 NA)
Total number of babies	2056	1038	1018
- Livebirth (%)	1740 (85%)	844 (81%)	896 (88%)
- Stillbirth (%)	261 (13%)	161 (16%)	100 (10%)
- Neonatal death (%)	49 (2%)	32 (3%)	17 (2%)
- Babies with unknown status (%)	6 (< 1%)	1 (< 1%)	5 (< 1%)

*NA* Not Available (from case records or birth register)

^a^Prolonged labour defined as no cervical dilatation over 4 h or lasting > 12 h in the active phase (i.e. from 4 cm)

The main reasons for transfer were prolonged/obstructed labour (Tanzania *n* = 40, 36%; Zambia *n* = 191, 51%), pre/eclampsia (Tanzania *n* = 12, 11%; Zambia *n* = 47, 12%), APH (Tanzania *n* = 11, 11%; Zambia *n* = 14, 4%) and labouring following previous caesarean section (Tanzania *n* = 6, 5%; Zambia *n* = 17, 5%). Other recorded reasons were sepsis, pre-term labour, cord prolapse, fetal distress, mental health issues, high parity, malaria, severe anaemia, and other health conditions. There was one case, in Tanzania, recorded as ‘unknown’.

Women most likely to be transferred were from Zambia, HIV positive, attended antenatal clinic < 4 times and living > 30 min away from the referral hospital (Table 2). Minor differences were observed between countries. Multiple imputed models specific to Tanzania and Zambia produced similar findings in most respects (Additional file 2), despite the need to impute 7.3% of data items (2.2% in Tanzania, 12.4% in Zambia). Both country-specific models found increased distance to referral hospital was associated with significantly increased odds of intrapartum transfer. Each model also found the same direction of association across all variables other than time of transfer and day of transfer. Time of admission showed opposite associations in Tanzania and Zambia. In Zambia, night admission was significantly associated with decreased odds of intrapartum transfer, while in Tanzania night admission was non-significantly associated with increased odds of transfer (Additional file 2).

**Table 2:** Comparison of characteristics of those with and without intrapartum transfer (percentages relate to known cases unless stated otherwise)

	Not Intrapartum transfer	Intrapartum Transfer	Multivariable OR (95% CI)
	***N*** = 1465	***N*** = 465	
Mean (SD) Mother’s age in years	27.8 (6.2)	25.9 (6.7)	^a^
Country			
Tanzania	855 (89%)	104 (11%)	1
Zambia	610 (63%)	361 (37%)	6.70 (4.61–9.73)
Married			
No	142 (68%)	68 (32%)	1
Yes	1315 (77%)	396 (23%)	0.74 (0.50–1.08)
**Unknown**	**8**	**1**	
Level of education			
None or primary only	611 (74%)	213 (26%)	1
Secondary	493 (82%)	110 (18%)	0.91 (0.68–1.21)
Higher or vocational	169 (85%)	30 (15%)	1.52 (0.79–2.91)
**Unknown**	**192**	**112**	
Formal Employment			
No	1301 (75%)	433 (25%)	1
Yes	152 (86%)	25 (14%)	0.57 (0.29–1.23)
**Unknown**	**12**	**7**	
Religion			
Christian	1266 (74%)	434 (26%)	1
Muslim or other^b^	193 (89%)	25 (11%)	1.05 (0.64–1.73)
**Unknown**	**6**	**6**	
Any previous stillbirth			
No	1407 (76%)	433 (24%)	1
Yes	52 (62%)	32 (38%)	1.12 (0.65–1.93)
**Unknown**	**6**		
HIV status			
Negative	1337 (77%)	410 (23%)	1
Positive	104 (69%)	46 (31%)	1.64 (1.07–2.51)
**Unknown**	**24**	**9**	
Care available at nearest health facility			
Basic EmOC	1203 (74%)	424 (26%)	1
First Aid	52 (80%)	13 (20%)	0.60 (0.27–1.34)
Comprehensive EmOC	185 (89%)	23 (11%)	0.65 (0.38–1.11)
**Unknown**	**25**	**5**	
Number of ANC visits			
≤ 4 visits	284 (70%)	119 (30%)	1
> 4 visits	917 (82%)	195 (18%)	0.48 (0.36–0.64)
**Unknown**	**264**	**151**	
Distance to nearest health facility			
< 30 min	1338 (78%)	369 (22%)	1
30–60 min	74 (52%)	69 (48%)	1.38 (0.87–2.17)
61–120 min	9 (39%)	14 (61%)	1.02 (0.37–2.80)
> 120 min	3 (30%)	7 (70%)	1.65 (0.28–9.54)
**Unknown**	**41**	**6**	
Distance to referral hospital			
< 30 min	704 (80%)	176 (20%)	1
30–60 min	645 (84%)	125 (16%)	2.30 (1.59–3.33)
61–120 min	55 (32%)	116 (68%)	9.80 (6.38–15.07)
> 120 min	53 (54%)	46 (46%)	5.32 (3.05–9.27)
**Unknown**	**8**	**2**	
Time of admission at referral hospital			
Day	832 (75%)	274 (25%)	1
Night	633 (77%)	191 (23%)	0.88 (0.68–1.12)
Day of admission to referral hospital			
Weekday	1056 (76%)	332 (24%)	1
Saturday or Sunday	408 (75%)	133 (25%)	0.94 (0.71–1.23)
**Unknown**	**1**	**0**	

Note: For categorical variables, the multivariable OR is the odds of intrapartum transfer for the given category, relative to the first category, adjusted for the effects of the other listed variables. Three singleton pregnancies were not included for missing intrapartum transfer status

^a^ A quadratic function was fitted for maternal age, providing linear and quadratic regression coefficients; this means that a single odds ratio for maternal age does not exist

^b^Only 4 of 193 ‘Muslim or other’ were not Muslim

A larger proportion of stillbirths (24% vs. 9%), neonatal (4% vs. 2%) and maternal deaths (3% vs. 1%) were each observed among those with intrapartum transfer than without (Table 3). There was also a considerably larger proportion of delay 2 among those who were transferred (32% vs. 7%).

**Table 3:** Outcomes of singleton pregnancy women grouped by intrapartum transfer status* – (percentages relate to known cases)

	Both Countries		Tanzania		Zambia	
	Not IT	IT	Not IT	IT	Not IT	IT
	N = 1465	N = 465	***N*** = 855	***N*** = 104	***N*** = 610	***N*** = 361
Status of baby						
Livebirth	1308 (89%)	334 (72%)	744 (87%)	38 (37%)	564 (92%)	296 (82%)
Stillbirth	129 (9%)	111 (24%)	90 (11%)	56 (54%)	39 (6%)	55 (15%)
Neonatal Death	28 (2%)	20 (4%)	21 (2%)	10 (10%)	7 (1%)	10 (3%)
Status of mother						
Alive	1452 (99%)	449 (97%)	849 (99%)	93 (89%)	603 (99%)	356 (99%)
Dead	13 (1%)	16 (3%)	6 (1%)	11 (11%)	7 (1%)	5 (1%)
Maternal near-miss						
No	828 (89%)	109 (54%)	797 (94%)	84 (82%)	31 (36%)	25 (25%)
Yes	103 (11%)	93 (46%)	49 (6%)	18 (18%)	54 (64%)	75 (75%)
**Unknown**	**534**	**263**	**9**	**2**	**525**	**261**
Delay 1						
No	582 (51%)	171 (61%)	337 (40%)	22 (23%)	254 (78%)	149 (81%)
Yes	569 (49%)	110 (39%)	496 (60%)	75 (77%)	73 (22%)	35 (19%)
**Unknown**	**314**	**184**	**22**	**7**	**292**	**177**
Delay 2						
No	1341 (93%)	310 (68%)	764 (92%)	73 (76%)	577 (95%)	237 (66%)
Yes	94 (7%)	144 (32%)	63 (8%)	23 (24%)	31 (5%)	121 (34%)
**Unknown**	**30**	**11**	**28**	**8**	**2**	**3**
Delay 3						
No	1272 (89%)	376 (83%)	705 (85%)	82 (87%)	567 (93%)	294 (82%)
Yes	164 (11%)	75 (17%)	123 (15%)	12 (13%)	41 (7%)	63 (18%)
**Unknown**	**29**	**14**	**27**	**10**	**2**	**4**

3 singleton pregnancies were not included due to missing intrapartum transfer status

### Qualitative

Eighty-five interviews were conducted; 37 in Tanzania and 48 in Zambia. Interviews lasted between 20 and 120 min. Demographic details of interviewees are in Table 4. Thirty-seven observations were conducted; 19 in Tanzania and 18 in Zambia. Median observation time was 70 min (range 15–240 min). In 16 cases women were observed being transferred to the referral centre, in 15 cases women were observed being received by the referral centre and in two cases the researcher observed the whole transfer.

**Table 4:** Demographic details of participants

	Tanzania			Zambia		
	Women *N* = 21	Partner *N* = 7	Health worker N = 9	Women N = 27	Partner N = 9	Heath worker N = 12
Age, median (range)	21 (18–41)	32 (26–50)	38 (25–53)	24 (18–45)	31 (25–56)	49 (33–65)
Marital status						
-Married	21	7	8	24	9	10
-Single	0	0	1	3	0	1
-Widowed	0	0	0	0	0	1
Education						
-Primary	5	1	0	9	3	0
-Secondary	15	6	0	15	4	0
-College	1	2	0	1	1	0
-Diploma	0	0	7	1	0	9
-Degree	0	1	1	1	1	3
-Other	0	0	1	0	0	1
Religion						
-Christian	21	7	9	27	26	12
-Muslim	0	0	0	0	1	0
Sampling group						
-Live birth	7	4		6	3	
-Stillbirth	10	5		13	3	
-Near miss	4	1		8	3	
Employment						
None/housewife	8	1	0	10	0	0
-Farmer	1	4	0	4	4	0
-Shop worker	3	1	0	5	1	0
-Tailor	3	0	0	4	0	0
-Clerical	4	0	0	3	1	0
-Business	1	4	0	1	3	0
-Nurse/midwife	1	0	4	0	0	7
-TBA/SMAG	0	0	3	0	0	3
-Ambulance driver	0	0	2	0	0	2

Three main categories were related to a core category, ‘journey of vulnerability*’*: *social threats to intrapartum transfers*, *barriers to timely care* and *reparative interventions for a positive experience;* these were consistent across countries*.*

#### Social threats to intrapartum transfers

Social threats to successful transfer were inter-related and included inadequate birth-preparedness, financial deprivation and geographical location.

### Inadequate birth preparedness

Birth preparedness constitutes preparation for labour, preparation to support the baby’s needs and awareness of danger signs that would require timely health facility referral^30^. When questioned about what birth preparedness meant to them, women focussed their answers on providing for the baby:

*‘The clinic health education class motivated us because during this class we are taught about the important of preparing in advance…such as buying baby layettes.’ [Woman, >1 stillbirth, Tanzania]*

Although some women mentioned observing for ‘danger signs’ they failed to provide detail of what these were and/or lacked understanding of the seriousness of them. For example, women stated that they needed to seek assistance if they were ‘leaking fluid from the vagina’ but would then wait several days to do so.

Birth preparedness is taught during antenatal clinics so relies on women’s attendance, however, there are multiple reasons why women fail to attend, as discussed by a TBA:

*‘There was no AIDS in the past, pregnant women did not have to take medicines so they get scared and decide to remain at home…some it is because they are not married…some because of distances, they can’t afford transport…husband refuses to give money’ [TBA, Tanzania]*

The woman’s position in her household made it difficult to prepare for any unexpected needs. One woman, in Zambia, who had experienced two stillbirths talked about how her husband would not assist in preparing for the birth, as he was ‘tired of having all these dead babies’. In Tanzania, health workers talked about the community pressures put on women to abstain from attending clinic:

*‘Some tribes think it is a taboo to attend clinic…some are prevented to attend by husbands and/or old parents who say for example, ‘we have delivered you all without clinics why now do you want to go for clinics?” [Health worker, Tanzania]*

Conversely, one partner talked openly about how the experiences of others had prompted him to support his wife’s preparation:

*‘I had a friend whose wife had pregnancy complications. She lost a lot of blood and became very weak. Looking for transport was a challenge too. It was almost too late by the time they got to the hospital. He nearly lost her. I reflected and decided that from now onwards I will be taking my wife to the hospital’* [Partner, Tanzania]

### Financial deprivation

A major reason for women not receiving timely intrapartum care was a lack of personal finances. One woman stated that she had been referred to the general hospital antenatally, however, despite having a previous caesarean section, did not have the money to attend. She talked about it being the norm for women to wait for active labour as this was the only way to get free transport:

*When a pregnant woman is referred not in labour, you have to find your transport to the referral Hospital. But when you are in labour, an ambulance is called to take you to the referral hospital. So most of the times, when a woman is referred, they wait to be in labour then go to the health centre so that transport can be provided. [Zambia, near-miss, live birth]*

Women were usually not in control of their own finances, making them reliant on their partners for support; this made it difficult for them to be ready for emergency situations:

*In our tribe men are the ones who stay with money. Most husbands do not give their wives adequate funds for the needs during delivery… [Tanzania, near-miss, live birth]*

Sometimes, despite a husband’s supportiveness, family resources were insufficient to support a transfer. One man, for example, talked about his own emotions when he was unable to transfer his wife comfortably:

*My experience was bad, we suffered moving from [name] rural health Centre to [name] rural health Centre, we struggled at night on a bicycle, it was a difficult journey. I feel bad ….. [Zambia, partner, live birth]*

### Geographical location

Although most participants acknowledged the benefits of tertiary facilities, the geographical location of their home was a major influence on their decision-making regarding place of birth. In the main, participants from rural areas feared intrapartum transfers and weighed up the negative aspects of transferring away from their villages.

*There are no good roads and also money to hire from town is a big challenge. Sometimes families have to contribute so that if the mother has been referred to another hospital then also provide escort and may need money for transport back. Sometimes there is no ambulance [Tanzania, postnatal woman, live birth]*

Health providers also commented on the distances that women need to travel when referred, stating that they were ‘too far’. Women’s locations also impacted on the drivers’ ability to transfer them swiftly. One ambulance driver stated:

*You can be allocated to collect a patient, you even start off but half way to the health facility you are called back to go and collect a more critical condition from a health facility in a different direction…. Most health facilities are in the remote areas and roads sometimes become impassable especially during the rainy season. Sometimes we get stuck due to bad roads and you just have to rely on help from the nearby villages. [Zambia, ambulance driver]*

Being transferred also had an impact on family members, who were unfamiliar with the surroundings and processes of the facility. One mother, whilst her daughter was being interviewed, talked about the impact of transfer on being able to fulfil cultural traditions:

*I was told by the nurse that my grandson had died and I needed to bury that child. I told the nurse that I was a stranger here and did not know anybody…finally, well-wishers who have relatives here helped me and we buried that child at a nearby village [Field notes: mother during woman’s interview, near-miss and stillbirth, Zambia]*

Observations revealed the fact that escorting family members were not treated well during transfer:

Relatives who came with the patient were not offered seats; they just stood in the corridor with the patient’s luggage. One of the relatives asked a maid who passed by for a seat, but they were told to go and sit outside. They stood in the corridor for 6 min and a security guard came and asked them to go to the mother’s shelter (a place where nursing relatives stay). They then informed the security guard that they were strangers and did not know the place but he instead told them to ask around and were shown the door. The 2 female relatives just walked away with the patient’s luggage.

Observation 9, Zambia.

#### Barriers to timely care

The main barriers to accessing timely care included insufficient transport facilities and personal reluctance.

### Insufficient transport facilities

A major barrier to timely referrals was the lack of available resources; ambulances were a particular problem. Even when an ambulance was available, insufficient fuel meant that women often had substantial delays before they could be transferred:

*I was told the baby wasn’t breathing and that she had been referred to [facility]. We were told to wait because the fuel in the ambulance wasn’t enough. We waited for a day. Then we were told to wait for other patients so that we can be taken together, that was bad. [Zambia, partner, stillbirth]*

It was common for women to be expected to make a payment towards the petrol if they needed to be transferred:

*It happens often for us to contribute petrol to ambulance. We think maybe the government has already provided ambulance so, it our responsibility to contribute for petrol … not less than fifty thousand [Tanzania shillings, 22 USD]. [Tanzania, Partner, live birth]*

Women had to either rely on the poor ambulance services available to them or find their own transport. One woman in labour with a breech presentation narrated her experience of attending 3 facilities before an accurate diagnosis was made. Her journey from a health post on an island to the referral hospital on the mainland took 32 h and involved public buses and a boat trip from a ‘good Samaritan’. She concluded by saying:

*I arrived at 15:00 hours and a few hours later I delivered a macerated female stillbirth… The referral experience was not good; we were not escorted by the water ambulance not even a nurse. We were just given a letter and to find our own transport…* [Zambia, woman, 2 stillbirths]

Observations confirmed the importance of the letter during transfer to the receiving nurse/midwife, who showed dissatisfaction when this was not issued. This often resulted in tensions between health providers. On one occasion this resulted in an argument between the referring and receiving nurse, in front of the woman. When being repeatedly asked for the referral letter, the referring nurse began to lose patience:

I did not come with a referral letter, I was just told to carry the patient. I quickly listened to the fetal heart, it was 90 beats per minute, I got the patient into the ambulance as I did not want to waste a lot of time to wait for the nurse to finish conducting a delivery and write a referral letter. In fact, I have to be quick because I have another patient in the ambulance who I need to take to the surgical department. I don’t want to waste a lot of time, if you don’t want this patient, I can take her back. A referral letter should not be an issue.

Observation 3, Zambia.

There are only two ambulances in each of the study settings; therefore poor availability was a major problem. However, some women questioned whether the ambulances were called for in good time:

*The nurse [in rural facility] called for an ambulance but it delayed because we were told that it was at another Centre referring another case…..The nurse was pulling the baby. She failed to deliver the head and the baby died on the vulva. If they sent me to the [District] hospital early enough, my child would have survived. [Zambia, woman, stillbirth]*

Others expressed anger at the multiple delays which they blamed for the death of their baby:

*The delay started from the health facility…..another delay was with the ambulance it came after 14:00hrs when the ambulance was called around 12:00hrs but the biggest delay was the delay to be taken to theatre. Had it not been for these delays I was going to have my baby. [Zambia, woman, stillbirth]*

### Personal reluctance

Personal reluctance to be transferred was related to either fear of receiving disrespectful care or not believing that the care they would receive would benefit themselves or their baby. The latter was related to believing it unnecessary to attend a facility if the baby’s death had already been diagnosed.

Many women were reluctant to be transferred to a facility that they deemed to be unwelcoming. They stated that they feared the health workers at the district facility who were often ‘harsh’ and would ‘shout’. For example, one woman said:

*You might find harsh nurse and you even ask God why you made me to meet this being. You even start creating fear during delivery….You find harsh nurse says why you women have come with dirty clothes without knowing other people don’t have soap to wash clothes…..you feel humiliated [Woman, Tanzania, live birth].*

Some women had heard ‘rumours that the nurses were not kind to women from the villages’ and were expecting poor care but, encouragingly, most women commented that the receiving facility welcomed them on arrival and treated them well. This was reinforced in the observations:

Escort nurse/midwife and the mother welcomed warmly. Escort nurse handed in the referral letter and explains about the anaemia with no blood transfusion facilities at [name] hospital. Receiving nurse was welcoming to the mother and relative…Relative and mother thanked the nurse and looked happier than before.

Observation 6, Tanzania.

One woman, who had birthed a stillborn baby previously, initially refused to be transferred from a local to a district facility because she was informed by the nurse that her twin babies had died; this implies that she considered the health of the babies the only reason to seek specialist care. She stated:

*I was told to be transferred to [name] district hospital but I refused because I was not ready. I felt like there was no reason why I should be transferred from one health facility to the other when my babies had died. [Zambia, woman, 2 stillbirths]*

However, a day later, and despite having two ultrasound scans, the woman gave birth to one stillborn baby and one live baby.

#### Reparative interventions for a positive experience

Social threats to successful transfer could be alleviated and barriers to care broken down, by reparative interventions, which included timely assessment, empathic communication and a positive outcome.

### Timely assessment

When women and partners believed that everything possible had been done to ensure they were transferred swiftly and assessed rapidly they were more accepting of the transfer. One woman, for example stated:

*I found a nurse who was attending to some patients. Immediately the stretcher I was on entered labor ward, she stopped attending to them and came to me. She greeted me with a smile…. [Tanzania, woman, live birth]*

Another woman, despite an arduous five hour journey, a ‘fear of transfer’ and a stillborn baby, stated:

*The transfer in itself wasn’t bad. When we arrived, they received us very well. I was taken on a stretcher to labour ward. The doctor came immediately. The health personnel were kind too….they were kind and respectful [Zambia, woman, stillbirth]*

Observations reinforced the fact that when received and assessed quickly, women and family members appeared less anxious and were more positive about their experience:

The woman and family appeared upset on arrival and were complaining about delays…the doctor came immediately and examined the woman. A blood transfusion was started…the mother and husband appeared more relaxed.

Observation 12, Tanzania.

### Empathic communication

Women valued being listened to and having a say in their care:

*I was received well; I was given a bed and examined….. They said I lost a lot of blood and recommended for a blood transfusion but I refused because of my religious beliefs…. they respected my views. I liked the care…. [Zambia, postnatal woman, near- miss]*

Similarly, partners welcomed being acknowledged and having their wives’ condition explained to them. Some discussed this as a redemptive process, whereby they went from fearing the facility to accepting it:

*At [name] Hospital, we were greeted when we just arrived. I was offered a seat and my wife was taken in the labour room to be examined. Later, I was explained the finding. I was also assured that my wife was in good hands, which was the most comforting thing….I was so scared when I was told about the referral, but the assurance I got when I arrived made my anxiety go. I’m still very grateful… [Zambia, partner, live birth]*

Most women were greeted well on arrival to the referral hospital, as observed:

It was a busy night. However, the midwives are calm and responding to relatives and client’s concerns accordingly. The midwife at the nurses’ desk greeted the mother to the client with a smile and respected her by offering her a seat. The receiving midwife offered a dignified care to the client by explaining every procedure to the client….All midwives and the Doctor handled the client with care and dignity.

Observation 3, Zambia.

### Positive outcome

Women who were transferred and gave birth to a live baby attributed this to a successful transfer, even if the care received was less than optimum. Some appeared to forgive negative aspects of care if it resulted in them having a healthy baby:

*At first the experience was not good. When we arrived the nurse shouted at me …. There were times some nurses could not even greet you, even when you ask something they would not even answer back….The health personnel managed to save both my life and that of my baby…. I’m so grateful (clapping her hands).* [Zambia, woman, near-miss]

Some women remained helpless; accepting whatever was done to them and believing a negative outcome to be part of ‘God’s Plan ’. Some women had a fatalistic attitude, suggesting that their experience and outcome was ‘out of their hands’. One partner reinforced this:

*We do not know about going to the clinic during pregnancies. All we know is farming and eating and to leave everything to God. [Tanzania, Partner, Live birth]*

However, several women talked about their experience being a ‘wake-up call’ which has made them determined to prepare more fully in future pregnancies. Women talked about ‘attending clinic’, ‘going early to the facility’ and ‘saving money for transport.’ One woman, despite giving birth to a stillborn baby, talked about how her experience of being transferred made her more resilient:

*I felt good because I got help where I was referred. I learned to always go forward and not turning back. I feel stronger and will do things differently next time [Tanzania, woman, stillbirth]*

## Discussion

For the first time, we have explored intrapartum transfers in low-income settings from multiple perspectives to detect factors that could make a difference to experiences and outcomes. Using a mixed-methods approach has enabled views and experiences to be placed in the wider social context. The quantitative findings, like others [4, 5], demonstrate clearly that transfers are an issue, leading to subsequent delays and poorer outcomes. The qualitative findings highlight the inefficient processes and resources that prevent timely intrapartum referral, including poor roads and limited transport, which are also reported elsewhere [7]. Addressing such findings are a challenge, which requires community involvement and government investment, however, we have also illuminated modifiable factors that, with minimal financial investment, could improve experiences and outcomes.

Qualitative findings suggest a level of vulnerability for women during transfer that resonates with the concept analysis described by Briscoe et al. [35]. Briscoe discovered three attributes to vulnerability directly related to childbirth: ‘threat’, ‘barrier’ and ‘repair’. For the first time, we have applied these attributes to research data to explore women’s transfer journeys (figure 1, Additional file 3). Briscoe identified different categories of threats: biological, psychological and social. The latter was most dominant in our data, creating the category: *social ‘threats’ to successful transfer*. Threats are those things that have the potential to cause harm, but can be anticipated in advance [36], thus identification of factors that increase the likelihood of transfers is the first step in developing strategies to ensure timely and effective care.

In relation to the social threats (inadequate birth-preparedness, financial deprivation and geographical location), synergies were evident between the quantitative and qualitative data. The quantitative data demonstrated that women who attend fewer antenatal appointments are more likely to be transferred. Women’s failure to attend antenatal clinic means that they did not receive advice to prepare for emergency situations. However, some attending women who received birth preparedness instruction lacked understanding of the rationale for signs and symptoms, resulting in them not seeking timely healthcare. The qualitative findings also highlighted why women fail to attend clinic, including fear of facility personnel, distance required to travel, lack of money for transport, lack of support from husband or community and HIV status. The latter point provides an explanation for our findings that women who are HIV positive are more likely to be transferred than those who are negative. Fear of revealing their HIV status prevents women receiving the antenatal care available which in turn diminishes opportunities for pregnancy screening and prevention of complications.

Those who lived over 30 min from the referral centre were more likely to be transferred. This is unsurprising as specialist services were unavailable in their immediate locality. The qualitative findings suggest that women in the rural villages feared being transferred to central facilities; barriers included being away from familiar surroundings, poor transport facilities, and personal reluctance. Poor transport is likely to have influenced the unacceptable rate of type 2 delays noted as 32% in the intrapartum transfer cases (Table 3). Delays were also a major cause of dissatisfaction amongst women and their families, and often blamed for the loss of a baby. Regrettably, it was reported that some women purposefully delayed their own referral in order to get free ambulance transfer, putting themselves and their baby at risk to prevent paying transport costs. Women anticipated ill-treatment by health professionals on arrival at the referral facility; this expectation was sometimes realised, although, on most occasions, women were well-received and treated respectfully. These redemptive experiences changed women’s and partners’ opinions of these facilities and could influence future decision-making. A positive experience with a good outcome was the most powerful reparative intervention, resulting in an increase in women’s resilience.

A number of reparative interventions were identified from our findings comprising timely assessment, empathic communication and a positive outcome. These interventions could reduce women’s vulnerability. Small actions made big differences, such as being welcomed with a smile, being included in decision-making and having family members acknowledged. Having a good experience and having a stillborn baby were not necessarily mutually exclusive; there were women who birthed a stillborn baby but suggested that their positive experience gave them a renewed strength to continue.

There were several limitations to this study. The case note review relied on data collected for other purposes and was often incomplete. Nevertheless, we imputed missing observations using multiple imputation, which is valid under assumptions that the reasons that data are missing are explained by information in the data set; although this is not a testable assumption, many of the significant effects detected were estimated to be substantial and so their importance (although not necessarily their magnitude) are very unlikely to be impacted by the substantial amount of missing data overall. Although the type of delay and reason for transfer was based on set criteria, it was assessed by individual researchers from the case notes and therefore type of delay is subjective. To minimise this, data was independently cross-checked with experienced clinicians and researchers and a consensus reached.

The stillbirth rate was higher than recorded national rates [20]. There are several possible explanations for this. This could be an anomaly, created by the period of data collection. Stillbirth data is challenging to obtain thus may be under-recorded, nationally; our study was adequately resourced enabling searching for missing case files. Alternatively it could be, as suggested by others [37] that stillbirth rates are much higher in rural and semi-rural settings, such as ours.

Although observations of transfers were made, it would have been useful to observe other aspects of care, such as birth preparedness instruction; the number of observations was restricted by available resources.

Despite these limitations, several recommendations can be made. Womens’ preparation for childbirth should move beyond a checklist of practical actions to ensure greater understanding of the urgency of accessing appropriate care at the right time. Individual planning of a woman’s childbirth journey should be facilitated, with recognition of potential and unique areas of vulnerability. Women should be supported to receive the recommended eight antenatal [38] contacts; this could be achieved, for example, through antenatal outreach services or group appointments. Steps should be taken to promote relationship building between the referring and receiving health provider; joint training and consistent policies should be provided. Women and family members should be welcomed on arrival at facilities and communicated with empathically.

## Conclusion

Intrapartum transfers are challenging in high-burden settings. However modifiable factors exist, which have the potential to improve experiences for women and their families and to ultimately improve maternal and newborn outcomes. Effective transfers rely on adequate resources and transport infrastructures but women also need to have the desire to attend the health facility and need to be empowered to do so. This study has demonstrated that women’s (and families) vulnerability can be reduced by empathic communication, timely assessment and a positive birth outcome; this can improve women’s resilience and influence decision-making in future pregnancies.

## Supplementary information

**Additional file 1.** Interview topic Guide.

**Additional file 2: Table 1.** Country-specific comparison of characteristics of those with and without intrapartum transfer.

**Additional file 3: Figure 1.** Conceptual Model of Journey of Vulnerability during intrapartum transfers.

## Data Availability

Data will be available upon reasonable request from the corresponding author. The full data set cannot be made public to maintain participant’s anonymity.

## Abbreviations

ANC: Antenatal clinic
CEI: Consumer engagement and involvement
CRF: Case review forms
EmONC: Emergency obstetric and neonatal care services
MICE: Multiple imputation by chained eqs.
NA: Not available
REDcap: Research Electronic Data capture
SMAG: Safe Motherhood Action Group
TBA: Traditional Birth attendant
WHO: World Health Organization
