# Supplementary material for: Journey of vulnerability: a mixed-methods study to understand intrapartum transfers in Tanzania and Zambia
Source: BMC Pregnancy Childbirth. 2020 May 14;20:292. doi: 10.1186/s12884-020-02996-8 (PMC7222428; doi:10.1186/s12884-020-02996-8)
Supplement: Supplementary file 1 — Additional file 1. Interview topic Guide. [file 12884_2020_2996_MOESM1_ESM.docx]

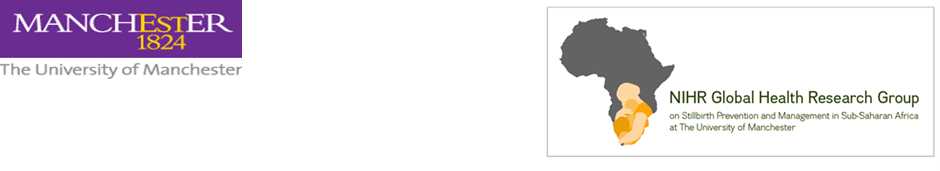


**Preventing stillbirth through timely intrapartum care: an exploratory mixed-methods study**: **Interview Topic Guide**

(Version 1, 28/03/18: Postnatal Women)

1. **Introduction, setting ground rules:**

***Introduce self, thank participant for taking part and confirm agrees to interview taking place. Ensure environment is comfortable. Discuss the following issues:***

- Review the nature and purpose of the research.
- No right or wrong answers, aim to understand experiences.
- Confidentiality, use of data.
- Explain the use of data recorder, transcription, use of pseudonym (invite to choose), use of verbatim quotes, will be taking field notes.
- Researcher aware that discussion might bring up difficult memories, explain can decline to answer any question or prompt; can ask to stop at any time if feels need to.
- Expected duration of interview.
- Check consent form signed, complete demographics questionnaire and EPDS.
- Ask if any questions.

***Check recorder working***

Introduce and switch on tape recorder

**General prompts**

- *Allow participant to respond uninterrupted and use open prompts if required to explore aspects of their experiences in depth.*
- *Can you tell me more about XXX?', 'What makes you say XXX?, How did XX make you feel?*

1. **Baseline details:**

|  | | | | | | | | | | Participant Code | | |
| --- | --- | --- | --- | --- | --- | --- | --- | --- | --- | --- | --- | --- |
| Age (years) |  | | | | | | | | | | | |
| Religion |  | | | | | | | | | | | |
| Country of birth |  | | | | | | | | | | | |
| County of residence |  | | | | | | | | | | | |
| Marital status | Single | Partner | | | Married | | | Widowed | | | | Separated/divorced |
| Employed | Full time | | Part-time | | | | | | No | | | |
| If employed, occupation |  | | | | | | | | | | | |
| Years of schooling |  | | | | | | | | | | | |
| Previous Births | Live Births | | | | | Stillbirths | | | | | Neonatal deaths | |
| Did you have any complications in your previous pregnancies? (If applicable)  (e.g. obstetric fistula) | No | Yes | | If yes, briefly describe treatment or care | | | | | | | | |
| Current birth | Live Birth | | | | | | Stillbirth | | | | | |
| Gestational age |  | | | | | | | | | | | |
| Mode of birth |  | | | | | | | | | | | |
| If Stillbirth, is the cause known | No  Yes, detail: | | | | | | | | | | | |

1. **Opening questions:**

- Can you tell us about your pregnancy?
  - Have you received any antenatal care, how often? By whom? Where?
  - Do you plan to attend antenatal care?
  - Why did you make this decision?
- Did you plan for this birth?
  - In what way?
- What influenced your planning?
  - People, information, media, community, other
- Did you feel prepared for the birth of this baby?
  - Yes, in what way? -transport, resources, support, access to health facility, knowledge of signs and symptoms
  - No, why not?
- Did you seek care during your pregnancy?
  - Who did you go to? What/who influenced your decision?
- What factors would encourage you to access care?
- What do you see as the barriers to you accessing care?
- Where did you give birth?
  - What/who has influenced this decision?
- What is your experience of maternity care/services?
  - Current pregnancy/previous pregnancy (if appropriate)/friends and family
  - Has this experience influenced how you feel about this pregnancy/birth?
  - How has this experience influenced your future decisions?
- If you could design the optimum care for pregnant women, what would this look like?
  - Environment/ Logistics/ Care provision/treatment/ Health professionals
- If you could have this pregnancy and birth all over again, what would you do differently, if anything?
- If you could give a message to health professionals, what would it be?
  - Positive or negative
- If you could give a close friend or relative advice about their pregnancy and birth, what would this be?

1. **This research**

- How do you feel about participating in this research?
- How do you feel about participating in this interview?
- Is there anything else you might want to add?

*At the close of the interview briefly summarise the main points to confirm interpretation with the participant. Ask if they wish to expand any responses or add anything else to the discussion. Thank the participants for their time.*

*Ask how they feel after talking about these experiences, do they want you to contact anyone? Family, friend, health worker?*

*Ensure participant has contact details for the local research team should they wish to discuss any aspect of the study.*

*Complete reflexive diary/field notes.*
