# Supplementary material for: Journey of vulnerability: a mixed-methods study to understand intrapartum transfers in Tanzania and Zambia
Source: BMC Pregnancy Childbirth. 2020 May 14;20:292. doi: 10.1186/s12884-020-02996-8 (PMC7222428; doi:10.1186/s12884-020-02996-8)
Supplement: Supplementary file 2 — Additional file 2: Table 1. Country-specific comparison of characteristics of those with and without intrapartum transfer. [file 12884_2020_2996_MOESM2_ESM.docx]

Table 1. Country-specific comparison of characteristics of those with and without intrapartum transfer (IT)

|  | | **Tanzania** | | |  | **Zambia** | | |
| --- | --- | --- | --- | --- | --- | --- | --- | --- |
|  | | **Not IT** | **IT** | **Multivariable OR (95% CI)** |  | **Not IT** | **IT** | **Multivariable OR (95% CI)** |
|  | | **N=855** | **N=104** |  |  | **N=610** | **N=361** |  |
| Mean (SD) mother’s age, in years | | 28.2 (5.5) | 28.9 (6.4) | ^#^ |  | 27.4 (7.0) | 25.1 (6.6) | ^#^ |
| Married | No | 76 | 13 | 1 |  | 66 | 55 | 1 |
|  | Yes | 771 | 91 | 0.82 (0.40-1.68) |  | 544 | 305 | 0.65 (0.40 – 1.06) |
|  | **Unknown** | **8** | **0** |  |  | **0** | **1** |  |
| Level of education | None or primary only | 402 | 60 | 1 |  | 209 | 153 | 1 |
|  | Secondary | 335 | 34 | 0.93 (0.57 – 1.50) |  | 158 | 76 | 0.88 (0.61 – 1.29) |
|  | Higher or vocational | 109 | 10 | 1.97 (0.81 – 4.80) |  | 60 | 20 | 1.37 (0.48 – 3.94) |
|  | **Unknown** | **9** | **0** |  |  | **183** | **112** |  |
| Formal Employment | No | 763 | 100 | 1 |  | 538 | 333 | 1 |
|  | Yes | 91 | 4 | 0.28 (0.08 – 0.94) |  | 61 | 21 | 0.96 (0.35 – 2.65) |
|  | **Unknown** | **1** | **0** |  |  | **11** | **7** |  |
| Religion | Christian | 659 | 79 | 1 |  | 607 | 355 |  |
|  | Muslim or other* | 192 | 24 | 1.03 (0.61 – 1.73) |  | 1 | 1 | ^##^ |
|  | **Unknown** | **4** | **1** |  |  | **2** | **5** |  |
| Any previous stillbirth | No | 838 | 101 | 1 |  | 569 | 332 | 1 |
|  | Yes | 16 | 3 | 1.17 (0.29 – 4.67) |  | 36 | 29 | 1.16 (0.63 – 2.13) |
|  | **Unknown** | **1** | **0** |  |  | **5** | **0** |  |
| HIV status | Negative | 814 | 94 | 1 |  | 523 | 316 | 1 |
|  | Positive | 31 | 10 | 4.07 (1.80 – 9.21) |  | 73 | 36 | 1.51 (0.92 – 2.49) |
|  | **Unknown** | **10** | **0** |  |  | **14** | **9** |  |
| Care available at nearest health facility | Basic EmOC | 646 | 80 | 1 |  | 557 | 344 | 1 |
|  | First Aid | 45 | 5 | 0.64 (0.23 – 1.83) |  | 7 | 8 | 0.62 (0.18 – 2.18) |
|  | Comprehensive EmOC | 139 | 14 | 0.67 (0.34 – 1.30) |  | 46 | 9 | 0.34 (0.15 – 0.79) |
|  | **Unknown** | **25** | **5** |  |  | **0** | **0** |  |
| Number of ANC visits | ≤ 4 visits | 227 | 59 | 1 |  | 57 | 60 | 1 |
|  | > 4 visits | 627 | 45 | 0.29 (0.19 – 0.46) |  | 290 | 150 | 0.76 (0.52 – 1.17) |
|  | **Unknown** | **1** | **0** |  |  | **263** | **151** |  |
| Distance home to nearest health facility | < 30 minutes | 770 | 97 | ^##^ |  | 568 | 272 | ^##^ |
|  | 30-60 minutes | 42 | 0 |  |  | 32 | 69 |  |
|  | 61-120 minutes | 3 | 0 |  |  | 6 | 14 |  |
|  | > 120 minutes | 1 | 1 |  |  | 2 | 6 |  |
|  | **Unknown** | **39** | **6** |  |  | **2** | **0** |  |
| Distance home to referral hospital | < 30 minutes | 181 | 21 | 1 |  | 523 | 155 | 1 |
|  | 30-60 minutes | 601 | 57 | 0.78 (0.42 – 1.42) |  | 44 | 68 | 4.69 (3.02 – 7.28) |
|  | 61-120 minutes | 30 | 14 | 3.67 (1.58 – 8.53) |  | 25 | 102 | 13.04 (7.92 – 21.46) |
|  | > 120 minutes | 36 | 11 | 2.45 (1.00 – 6.01) |  | 17 | 35 | 7.36 (3.74 – 14.49) |
|  | **Unknown** | **7** | **1** |  |  | **1** | **1** |  |
| Time of admission to referral hospital | Day | 534 | 58 | 1 |  | 298 | 216 | 1 |
|  | Night | 321 | 46 | 1.40 (0.89 – 2.19) |  | 312 | 145 | 0.71 (0.52 – 0.96) |
| Day of admission to referral hospital | Week day | 642 | 76 | 1 |  | 414 | 256 | 1 |
|  | Saturday or Sunday | 213 | 28 | 1.15 (0.70 – 1.89) |  | 195 | 105 | 0.92 (0.66 – 1.29) |
|  | **Unknown** | **0** | **0** |  |  | **1** | **0** |  |

Note: For categorical variables, the multivariable OR is the odds of intrapartum transfer for the given category, relative to the first category, adjusted for the effects of the other listed variables. Three singleton pregnancies were not included for missing intrapartum transfer status.

^#^ A quadratic function was fitted for maternal age, providing linear and quadratic regression coefficients; this means that a single odds ratio for maternal age does not exist.

*Only 4 of 192 ‘Muslim or other’ in Tanzania were not Muslim.

^##^ Distance home to nearest health facility was not included in either country-specific model due to small category size. Religion was not included in the Zambia-specific model for the same reason.
