# Supplementary material for: Journey of vulnerability: a mixed-methods study to understand intrapartum transfers in Tanzania and Zambia
Source: BMC Pregnancy Childbirth. 2020 May 14;20:292. doi: 10.1186/s12884-020-02996-8 (PMC7222428; doi:10.1186/s12884-020-02996-8)
Supplement: Supplementary file 3 — Additional file 3: Figure 1. Conceptual Model of Journey of Vulnerability during intrapartum transfers. [file 12884_2020_2996_MOESM3_ESM.docx]

Figure 1. Conceptual model of Journey of Vulnerability during intrapartum transfers

Satisfaction

Increased resilience

Dissatisfaction

Increased vulnerability

Threats resolved:

Timely transfer/care

Respectful care

Positive outcomes

Threats not resolved:

Delayed transfer/care

Disrespectful care

Poor outcomes

**Social Complexity**
